# *Mycobacterium bovis* in a bull on a university farm: public health implications

**DOI:** 10.11604/pamj.2020.37.40.21187

**Published:** 2020-09-09

**Authors:** Monsuru Oladunjoye Tijani, Hezekiah Kehinde Adesokan, Olajide Babatunde Kasali, Simeon Idowu Cadmus

**Affiliations:** 1 Department of Veterinary Pathology, University of Ibadan, Ibadan, Nigeria,; 2 Department of Veterinary Public Health and Preventive Medicine, University of Ibadan, Ibadan, Nigeria,; 3 Center for Control and Prevention of Zoonoses, University of Ibadan, Ibadan, Nigeria

**Keywords:** Bovine tuberculosis, epidemiology, public health

## Abstract

An unsuspected bull in a private herd of forty cattle heads in south-western Nigeria died suddenly following three days´ treatment against tick infestation. Post-mortem findings revealed multi-focal widespread nodules in all lobes of the lungs with markedly enlarged lymph nodes. Isolate from cultured sample was subjected to spoligotyping which confirmed the isolate as Mycobacterium bovis (M. bovis) belonging to the SB1027 clade with octal number 676773776277600 (Figure 1). This finding has implications on the health of the cattle handlers considering aerosol inhalation of disseminated bacilli from the lungs of the infected bull through cough sprays. Routine screening of cattle for tuberculosis is therefore emphasized.

## Introduction

*Mycobacterium bovis* is the primary cause of tuberculosis (TB) infection in cattle and could infect humans through aerosol inhalation and consumption of unpasteurized milk. While TB in humans as a result of *M. bovis* infection is relatively rare in comparison to *M. tuberculosis*, it remains a cause for concern in persons at high risk, such as cattle handlers and abattoir workers [1, 2]. Human infection due to *M. bovis* commonly occurs through consumption of unpasteurised milk and milk products; however, aerosol dissemination as a result of pulmonary disease is also known [2]. There is increasing risk of exposure to this disease by humans as only a few doses are required to elicit infection. We report a case of *M. bovis* infection in a bull among a herd of cattle on a university farm in south-western Nigeria with attendant public health implications to the handlers and exposed students.

## Patient and observation

**Case study:** *M. bovis* was isolated from a four-year-old Sokoto Gudali bull from a herd of forty cattle on a university farm in south-western Nigeria. The bull was observed to be anorexic, lethargic and recumbent, three days prior to its death. Physical examination before death revealed presence of numerous engorged ticks on the skin, congested and moist mucous membranes, weak pulse, tachypnoea and a rectal temperature of 39°C. The animal had previously been treated with diminazene aceturate and albendazole. The animal died suddenly, three days following the observation of the clinical signs.

**Figure 1:**
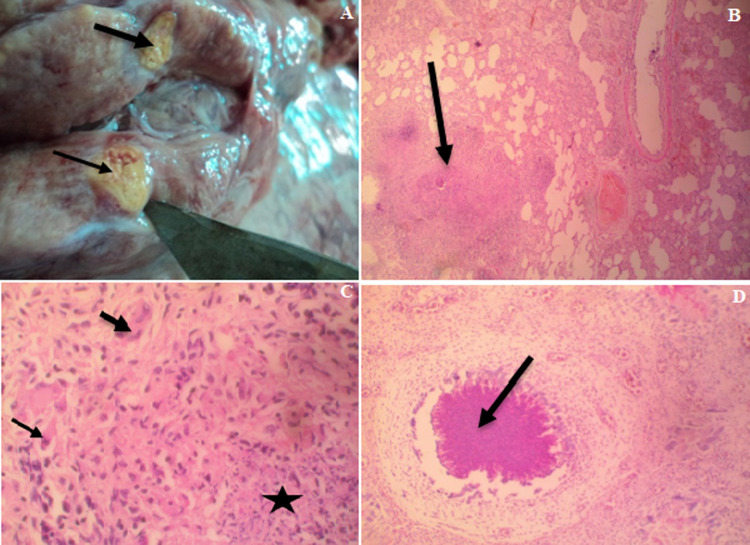
A) cut surface of the mediastinal lymph node showing yellowish, hard nodules (arrows); B) photomicrograph of the lung showing a large granuloma (arrow), X40, H&E; C) photomicrograph of a lung nodule showing the centre of caseous necrosis (black star) surrounded by lymphocytes and macrophages with a few multinucleated giant cells (arrows) at the periphery of the lesion, X400, H&E; D) photomicrograph of a splenic nodule showing a granuloma with a centre of caseous necrosis (arrow) x100, H&E

**Gross pathology:** at postmortem, the most significant finding was the presence of multifocal widespread nodules (1-3.5 cm in diameter) in all lobes of the lungs. The nodules were yellow and were either firm or hard and gritty (Figure 1A).

**Cytology:** microscopic examination of Ziehl-Nielsen-stained imprints of the nodules from the lungs, submandibular and mediastinal lymph nodes revealed numerous pink, short acid-fast rods.

**Histopathology:** the lungs contained multiple discrete to coalescing granulomata, each consisting of a central area of caseous necrosis surrounded by lymphocytes, macrophages and numerous Langhans and foreign body giant cells (Figure 1B, C, D). Occasionally, the caseous centres of the granulomata were mineralized. The granulomata were often surrounded by variable amount of fibrous connective tissues. Most of the alveoli contained numerous macrophages, occasionally trapped within pinkish (oedematous) fluid. Similar granulomata were observed in the spleen and lymph nodes.

**Cultural isolation:** nodular lesions on the lungs and granulomatous mediastinal lymph nodes were processed following an earlier described procedure [3]. The concentrate thus obtained was inoculated onto Löwenstein-Jensen slopes with pyruvate and/or glycerol and incubated at 37°C for 12 weeks. A positive growth was harvested and subjected to deletion typing to identify *M. tuberculosis* complex (MTC) isolate by the polymerase chain reaction (PCR) amplification of genomic regions of difference (RD), as described elsewhere [4]. The isolate was identified as a member of MTC and was thereafter processed by spoligotyping following a standardized method [5] using a commercially available kit (Isogen Biosciences BV, Maarsen, The Netherlands). *Mycobacterium tuberculosis* H37Rv, *M. bovis* Bacille Calmette-Guérin and sterile distilled water were used as controls. Resulting spoligotype pattern was compared to existing patterns in an international spoligotyping database profile (SITVIT2) [6] according to Couvin *et al*. [7]. A spoligotype family was assigned as previously described [6].

## Discussion

The spoligotyping confirmed the isolate as *M. bovis* belonging to the SB1027 clade with octal number 676773776277600. This isolated *M. bovis* strain belonging to SB1027 from the case study has been previously reported in cattle in Nigeria and neighbouring African country, Chad [8]. This strain is one of the most abundant strains in cattle in Nigeria [8] and may have thus become well adapted to the environment and resistant to host mechanism. From this study, the strain was isolated from the lungs of the infected bull. The animal may therefore have been shedding the organism through aerosols to the environment, thus potentiating the survivability and transmission chain of the organism. As the lung is the main site of TB in cattle; farmers, veterinarians and other workers in close contact with diseased animals are principally affected by the inhalation route [9].

Reports have shown that infection of the occupationally exposed individuals with *M. bovis* is more likely through the aerogenous route rather than through the conventional oral route. According to Muller *et al*. [10], the occurrence of zoonotic TB is greatly dependent on the presence of TB in cattle. The finding of this study therefore has significant public health implications considering the use of such an unrecognised infected bull for teaching purposes in a university setting. This portends potentially undetected TB transmission to exposed individuals who were in regular contacts with the animal. Human TB due to *M. bovis* can certainly be as severe as that due to *M. tuberculosis*. In fact, data from San Diego, California, USA, revealed that persons with *M. bovis* were 2.55 times more likely to die during treatment than those with *M. tuberculosis* [11]. More so, strains of *M. bovis* are intrinsically resistant to pyrazinamide with implications on TB control.

## Conclusion

We reported a case of cattle infected with *M. bovis* belonging to the SB1027 among cattle herds in a university setting and this has attendant public health implications for handlers and students on exposure to it during training. It is therefore of paramount importance that routine screening for bovine TB particularly in settings characterized with regular contacts with cattle be made mandatory. This will go a long way to safe guard the health of the animals, the exposed individuals as well as the general public.

## References

[1] Smith RM, Drobniewski F, Gibson A, Montague JD, Logan MN, Hunt D (2004). *Mycobacterium bovis* infection, United Kingdom. Emerg Infect Dis.

[2] Cadmus S, Palmer S, Okker M, Dale J, Gover K, Smith N (2006). Molecular analysis of human and bovine tubercle bacilli from a local setting in Nigeria. J Clin Microbiol.

[3] Becton Dickinson (1999). Becton Dickinson BBL™ Mycoprep™ Specimen digestion/decontamina tion kit manual for processing of mycobacterial specimens.

[4] Warren RM, Gey van Pittius NC, Barnard M, Hesseling A, Engelke E, De Kock M (2006). Differentiation of *Mycobacterium tuberculosis* complex by PCR amplification of genomic regions of difference. Int J Tuberc Lung Dis.

[5] Kamerbeek J, Schouls L, Kolk A, Van Agterveld M, Van Soolingen D, Kuijper S (1997). Simultaneous detection and strain differentiation of *Mycobacterium tuberculosis* for diagnosis and epidemiology. J Clin Microbiol.

[6] Karine Brudey, Jeffrey Driscoll R, Leen Rigouts, Wolfgang Prodinger M, Andrea Gori, Sahal Al-Hajoj A (2006). Mycobacterium tuberculosis, complex genetic diversity: mining the fourth international spoligotyping database (SpolDB4) for classification, population genetics and epidemiology. BMC Microbiol.

[7] Couvin D, David A, Zozio T, Rastogi N (2019). Macro-geographical specificities of the prevailing tuberculosis epidemic as seen through SITVIT2, an updated version of the *Mycobacterium tuberculosis* genotyping database. Infect Genet Evol.

[8] Borna Müller, Markus Hilty, Stefan Berg, Carmen Garcia-Pelayo M, James Dale, Laura Boschiroli M (2009). African 1, an epidemiologically important clonal complex of *Mycobacterium bovis* dominant in Mali, Nigeria, Cameroon, and Chad. J Bacteriol.

[9] Shaukat Bilal, Mudassir Iqbal, Philip Murphy, Joan Power (2010). Human bovine tuberculosis: remains in the differential. J Med Microbiol.

[10] Borna Müller, Salome Dürr, Silvia Alonso, Jan Hattendorf, Cláudio Laisse JM, Sven Parsons DC (2013). *Zoonotic Mycobacterium bovis* induced tuberculosis in humans. Emerg Infect Dis.

[11] Timothy Rodwell C, Marisa Moore, Kathleen Moser S, Stephanie Brodine K, Steffanie Strathdee A (2008). Tuberculosis from *Mycobacterium bovis* in binational communities, United States. Emerg Infect Dis.

